# Supplementary material for: Extensible Immunofluorescence (ExIF) accessibly generates high-plexity datasets by integrating standard 4-plex imaging data
Source: Nat Commun. 2025 May 17;16:4606. doi: 10.1038/s41467-025-59592-7 (PMC12085645; doi:10.1038/s41467-025-59592-7)
Supplement: Supplementary file 1 — Supplementary Information [file 41467_2025_59592_MOESM1_ESM.pdf]

Supplementary

Supplementary Table 1. List of per cell features and feature categories measured via CellProfiler.

**Morphology Features**	**Radial Distribution Features**	**Texture Features**
1. AreaShape_Area	1. RadialDistribution_FracAtD_1of5	1. Texture_AngularSecondMoment_3_00_256
2. AreaShape_Compactness	2. RadialDistribution_FracAtD_2of5	2. Texture_AngularSecondMoment_3_01_256
3. AreaShape_Eccentricity	3. RadialDistribution_FracAtD_3of5	3. Texture_AngularSecondMoment_3_02_256
4. AreaShape_EquivalentDiameter	4. RadialDistribution_FracAtD_4of5	4. Texture_AngularSecondMoment_3_03_256
5. AreaShape_Extent	5. RadialDistribution_FracAtD_5of5	5. Texture_Contrast_3_00_256
6. AreaShape_FormFactor	6. RadialDistribution_MeanFrac_1of5	6. Texture_Contrast_3_01_256
7. AreaShape_MajorAxisLength	7. RadialDistribution_MeanFrac_2of5	7. Texture_Contrast_3_02_256
8. AreaShape_MaxFeretDiameter	8. RadialDistribution_MeanFrac_3of5	8. Texture_Contrast_3_03_256
9. AreaShape_MaximumRadius	9. RadialDistribution_MeanFrac_4of5	9. Texture_Correlation_3_00_256
10. AreaShape_MeanRadius	10. RadialDistribution_MeanFrac_5of5	10. Texture_Correlation_3_01_256
11. AreaShape_MedianRadius	11. RadialDistribution_RadialCV_1of5	11. Texture_Correlation_3_02_256
12. AreaShape_MinFeretDiameter	12. RadialDistribution_RadialCV_2of5	12. Texture_Correlation_3_03_256
13. AreaShape_MinorAxisLength	13. RadialDistribution_RadialCV_3of5	13. Texture_DifferenceEntropy_3_00_256
14. AreaShape_Perimeter	14. RadialDistribution_RadialCV_4of5	14. Texture_DifferenceEntropy_3_01_256
15. AreaShape_Solidity	15. RadialDistribution_RadialCV_5of5	15. Texture_DifferenceEntropy_3_02_256
16. Neighbors_NumberOfNeighbors_Adjacent	16. RadialDistribution_ZernikeMagnitude_0_0	16. Texture_DifferenceEntropy_3_03_256
17. Neighbors_PercentTouching_Adjacent	17. RadialDistribution_ZernikeMagnitude_1_1	17. Texture_DifferenceVariance_3_00_256
	18. RadialDistribution_ZernikeMagnitude_2_0	18. Texture_DifferenceVariance_3_01_256
	19. RadialDistribution_ZernikeMagnitude_2_2	19. Texture_DifferenceVariance_3_02_256
**Intensity Features**	20. RadialDistribution_ZernikeMagnitude_3_1	20. Texture_DifferenceVariance_3_03_256
	21. RadialDistribution_ZernikeMagnitude_3_3	21. Texture_Entropy_3_00_256
1. Intensity_IntegratedIntensityEdge	22. RadialDistribution_ZernikeMagnitude_4_0	22. Texture_Entropy_3_01_256
2. Intensity_IntegratedIntensity	23. RadialDistribution_ZernikeMagnitude_4_2	23. Texture_Entropy_3_02_256
3. Intensity_LowerQuartileIntensity	24. RadialDistribution_ZernikeMagnitude_4_4	24. Texture_Entropy_3_03_256
4. Intensity_MADIntensity	25. RadialDistribution_ZernikeMagnitude_5_1	25. Texture_InfoMeas1_3_00_256
5. Intensity_MassDisplacement	26. RadialDistribution_ZernikeMagnitude_5_3	26. Texture_InfoMeas1_3_01_256
6. Intensity_MaxIntensityEdge	27. RadialDistribution_ZernikeMagnitude_5_5	27. Texture_InfoMeas1_3_02_256
7. Intensity_MaxIntensity	28. RadialDistribution_ZernikeMagnitude_6_0	28. Texture_InfoMeas1_3_03_256
8. Intensity_MeanIntensityEdge	29. RadialDistribution_ZernikeMagnitude_6_2	29. Texture_InfoMeas2_3_00_256
9. Intensity_MeanIntensity	30. RadialDistribution_ZernikeMagnitude_6_4	30. Texture_InfoMeas2_3_01_256
10. Intensity_MedianIntensity	31. RadialDistribution_ZernikeMagnitude_6_6	31. Texture_InfoMeas2_3_02_256
11. Intensity_MinIntensityEdge	32. RadialDistribution_ZernikeMagnitude_7_1	32. Texture_InfoMeas2_3_03_256
12. Intensity_MinIntensity	33. RadialDistribution_ZernikeMagnitude_7_3	33. Texture_InverseDifferenceMoment_3_00_256
13. Intensity_StdIntensityEdge	34. RadialDistribution_ZernikeMagnitude_7_5	34. Texture_InverseDifferenceMoment_3_01_256
14. Intensity_StdIntensity	35. RadialDistribution_ZernikeMagnitude_7_7	35. Texture_InverseDifferenceMoment_3_02_256
15. Intensity_UpperQuartileIntensity	36. RadialDistribution_ZernikeMagnitude_8_0	36. Texture_InverseDifferenceMoment_3_03_256
	37. RadialDistribution_ZernikeMagnitude_8_2	37. Texture_SumAverage_3_00_256
	38. RadialDistribution_ZernikeMagnitude_8_4	38. Texture_SumAverage_3_01_256
	39. RadialDistribution_ZernikeMagnitude_8_6	39. Texture_SumAverage_3_02_256
	40. RadialDistribution_ZernikeMagnitude_8_8	40. Texture_SumAverage_3_03_256
	41. RadialDistribution_ZernikeMagnitude_9_1	41. Texture_SumEntropy_3_00_256
	42. RadialDistribution_ZernikeMagnitude_9_3	42. Texture_SumEntropy_3_01_256
	43. RadialDistribution_ZernikeMagnitude_9_5	43. Texture_SumEntropy_3_02_256
	44. RadialDistribution_ZernikeMagnitude_9_7	44. Texture_SumEntropy_3_03_256
	45. RadialDistribution_ZernikeMagnitude_9_9	45. Texture_SumVariance_3_00_256
	46. RadialDistribution_ZernikePhase_0_0	46. Texture_SumVariance_3_01_256
	47. RadialDistribution_ZernikePhase_1_1	47. Texture_SumVariance_3_02_256
	48. RadialDistribution_ZernikePhase_2_0	48. Texture_SumVariance_3_03_256
	49. RadialDistribution_ZernikePhase_2_2	49. Texture_Variance_3_00_256
	50. RadialDistribution_ZernikePhase_3_1	50. Texture_Variance_3_01_256
	51. RadialDistribution_ZernikePhase_3_3	51. Texture_Variance_3_02_256
	52. RadialDistribution_ZernikePhase_4_0	52. Texture_Variance_3_03_256
	53. RadialDistribution_ZernikePhase_4_2	
	54. RadialDistribution_ZernikePhase_4_4	
	55. RadialDistribution_ZernikePhase_5_1	
	56. RadialDistribution_ZernikePhase_5_3	
	57. RadialDistribution_ZernikePhase_5_5	
	58. RadialDistribution_ZernikePhase_6_0	
	59. RadialDistribution_ZernikePhase_6_2	
	60. RadialDistribution_ZernikePhase_6_4	
	61. RadialDistribution_ZernikePhase_6_6	
	62. RadialDistribution_ZernikePhase_7_1	
	63. RadialDistribution_ZernikePhase_7_3	
	64. RadialDistribution_ZernikePhase_7_5	
	65. RadialDistribution_ZernikePhase_7_7	
	66. RadialDistribution_ZernikePhase_8_0	
	67. RadialDistribution_ZernikePhase_8_2	
	68. RadialDistribution_ZernikePhase_8_4	
	69. RadialDistribution_ZernikePhase_8_6	
	70. RadialDistribution_ZernikePhase_8_8	
	71. RadialDistribution_ZernikePhase_9_1	
	72. RadialDistribution_ZernikePhase_9_3	
	73. RadialDistribution_ZernikePhase_9_5	
	74. RadialDistribution_ZernikePhase_9_7	
	75. RadialDistribution_ZernikePhase_9_9	

Supplementary Table 2. List of antibodies and cell labelling reagents used.

	Species	Dilution	Supplier	Catalogue number	Method
**Primary antibodies**					
CD44	Mouse	1:500	Cell Signaling Technology	3570S	Indirect IF
CD44std	Mouse	1:500	Thermo-Fischer Scientific	BMS113	Indirect IF
CD44v9	Mouse	1:500	Novus Biologicals	NBP2-53204	Indirect IF, 4i
COX IV	Mouse	1:500	Abcam	ab33985	Indirect IF, 4i
E-Cadherin	Mouse	1:500	BD Biosciences	610181	Indirect IF
EpCAM	Mouse	1:500	Cell Signaling Technology	2929S	Indirect IF
Fibrillarin	Rabbit	1:500	Abcam	ab5821	Indirect IF, 4i
GM130	Mouse	1:500	BD Biosciences	610823	Indirect IF, 4i
N-Cadherin	Mouse	1:200	Cell Signaling Technology	14215S	Indirect IF
NF-kB p65	Rabbit	1:500	Cell Signaling Technology	8242S	Indirect IF, 4i
PTEN	Mouse	1:500	Thermo-Fischer Scientific	32-5800	Indirect IF
Vimentin	Mouse	1:500	Abcam	ab8978	Indirect IF
α-Tubulin	Mouse	1:500	Abcam	ab7291	Indirect IF
β-catenin	Rabbit	1:500	Thermo-Fischer Scientific	71-2700	Indirect IF, 4i
**Fluorescent Labels**					
4’, 6-diamidino-2-phenylindole (DAPI)	N/A	1:2000 (Indirect IF, 4i), 1:4000 (CycIF)	Sigma-Aldrich Pty Ltd	D9532	Indirect IF, 4i, CycIF
Atto-647 Phalloidin	N/A	1:2000	Atto-Tec GmbH	AD647-81	Indirect IF, 4i,
**Secondary antibodies**					
Anti-mouse IgG (H+L), F(ab’)2 AF 488	Goat	1:1000	Cell Signaling Technology	448S	Indirect IF, 4i
Anti-rabbit IgG (H+L), F(ab’)2 AF555	Goat	1:1000	Cell Signaling Technology	4413S	Indirect IF, 4i
**Conjugated antibodies**					
E-Cadherin AF488	N/A	1:200	Cell Signaling Technology	3199S	CycIF
ZEB1 AF488	N/A	1:500	Cell Signaling Technology	29153S	CycIF
N-Cadherin AF488	N/A	1:200	Cell Signaling Technology	81673S	CycIF
Pan-cytokeratin AF488	N/A	1:500	Thermo-Fischer Scientific	53-9003-80	CycIF
Actin AF555	N/A	1:500	Abcam	ab179467	CycIF
β-catenin (In-house conjugation) AF555	N/A	1:200	Thermo-Fischer Scientific	71-2700 A20187	CycIF
EpCAM AF555	N/A	1:500	Cell Signaling Technology	5488S	CycIF
CD44 PE	N/A	1:500	Cell Signaling Technology	8724S	CycIF
β-tubulin AF 647	N/A	1:500	Cell Signaling Technology	3624S	CycIF
ZO-1 AF647	N/A	1:500	Cell Signaling Technology	98225S	CycIF
pSMAD2/pSMAD3 AF647	N/A	1:200	BD Biosciences	562696	CycIF
Vimentin AF647	N/A	1:500	BioLegend	677807	CycIF

Supplementary Table 3. Summary of processed datasets used.

Dataset Name	# Patches per well	# Wells	Patch resolution	Multiplexed	Markers	# Markers	Treatments
4i	583	1	256x256	Yes	α-Tubulin<br>DNA<br>CoxIV<br>Fibrillarin<br>GM130<br>F-Actin<br>β-Catenin<br>NF-κB	8	None
ExIF EMT	296	24	256x256	No	DNA<br>F-Actin<br>β-catenin<br>E-Cadherin<br>EpCAM<br>N-Cadherin<br>PTEN<br>Vimentin<br>CD44total<br>CD44std<br>CD44v9	11	Control<br>EGF<br>TGF-β1
Multiplexed EMT	576	30	256x256	Yes	DNA<br>Actin<br>β-catenin<br>β-tubulin<br>CD44<br>E-Cadherin<br>EpCAM<br>N-Cadherin<br>pan-cytokeratin<br>pSMAD23<br>Vim<br>Zeb1<br>ZO1	13	Control<br>EGF<br>TGF-β1

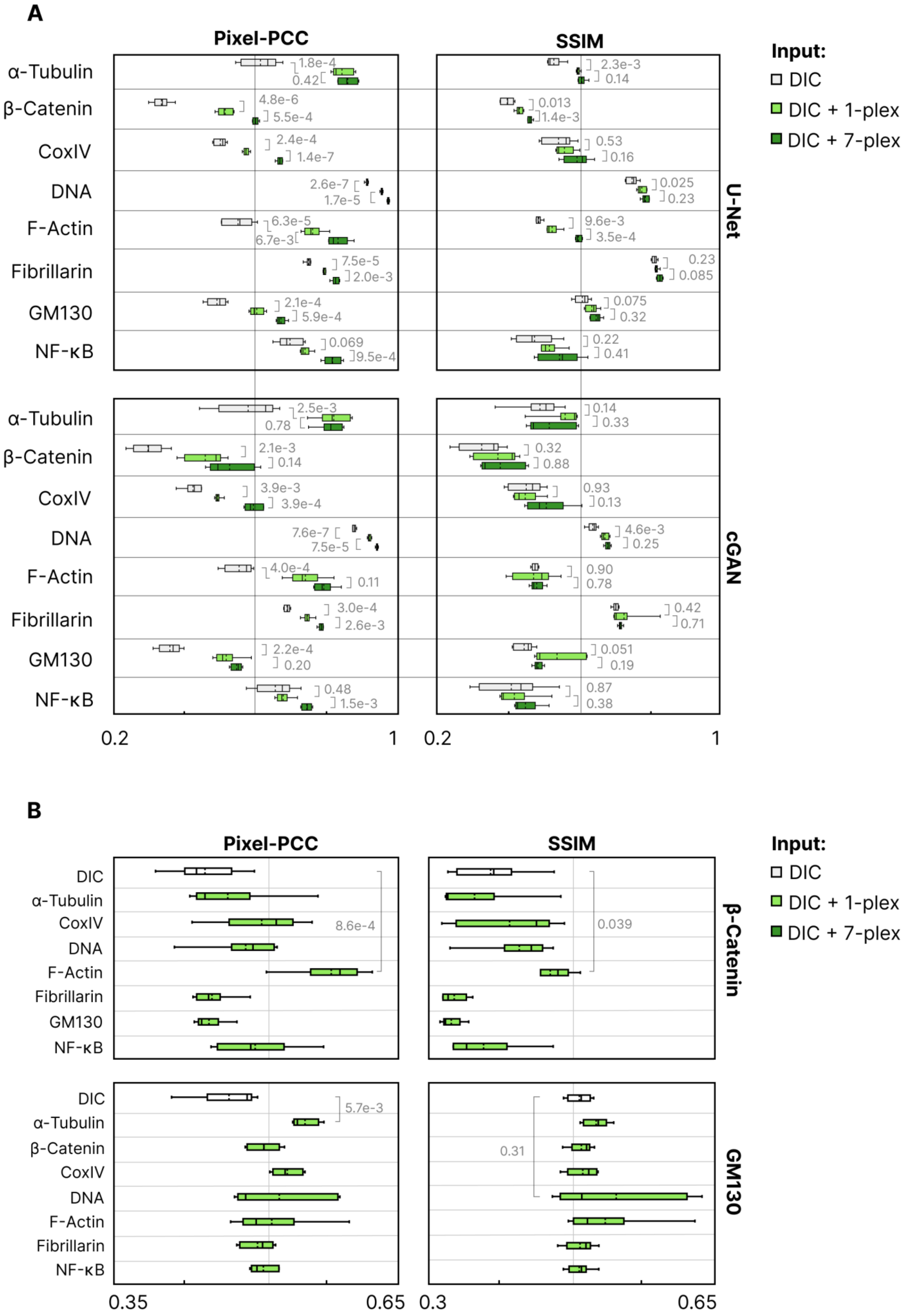

A
Pixel-PCC
SSIM
Input:
DIC
DIC + 1-plex
DIC + 7-plex
α-Tubulin
β-Catenin
CoxIV
DNA
F-Actin
Fibrillarin
GM130
NF-κB
U-Net
cGAN
1.8e-4
0.42
4.8e-6
5.5e-4
2.4e-4
1.4e-7
2.6e-7
1.7e-5
6.3e-5
6.7e-3
7.5e-5
2.0e-3
2.1e-4
5.9e-4
0.069
9.5e-4
2.3e-3
0.14
0.013
1.4e-3
0.53
0.16
0.025
0.23
9.6e-3
3.5e-4
0.23
0.085
0.075
0.32
0.22
0.41
2.5e-3
0.78
2.1e-3
0.14
3.9e-3
3.9e-4
7.6e-7
7.5e-5
4.0e-4
0.11
3.0e-4
2.6e-3
2.2e-4
0.20
0.48
1.5e-3
0.14
0.33
0.32
0.88
0.93
0.13
4.6e-3
0.25
0.90
0.78
0.42
0.71
0.051
0.19
0.87
0.38
0.2
1
0.2
1
B
Pixel-PCC
SSIM
Input:
DIC
DIC + 1-plex
DIC + 7-plex
DIC
α-Tubulin
CoxIV
DNA
F-Actin
Fibrillarin
GM130
NF-κB
8.6e-4
0.039
β-Catenin
DIC
α-Tubulin
β-Catenin
CoxIV
DNA
F-Actin
Fibrillarin
NF-κB
5.7e-3
0.31
GM130
0.35
0.65
0.3
0.65

Supplementary Figure 1. *(**A**) Median image metric (titled) scores across 5 cross-validated folds for architecture as labelled on the right. Different input schemes shown in colour with only the best performing pair for DIC+1-plex shown. Remaining DIC+1-plex performance shown in supplementary figure 1B. (**B**) Image metric (titled) scores across 5 cross-validated folds for given categories for β-Catenin (top) or GM130 (bottom), comparing ResViT models with DIC only input (grey) or DIC plus 1 fluorescence channel (light green; additional fluorescence channel denoted on left axis). Self-prediction (where fluorescence inputs and targets are identical) was avoided. All box plots depict data distributions as follows: dashed center line, median; solid center line, mean; box limits, upper and lower quartiles; whiskers, 1.5x interquartile range. Significance testing reflects Welsh's two-sided t-test. Source data provided as detailed in data availability.*

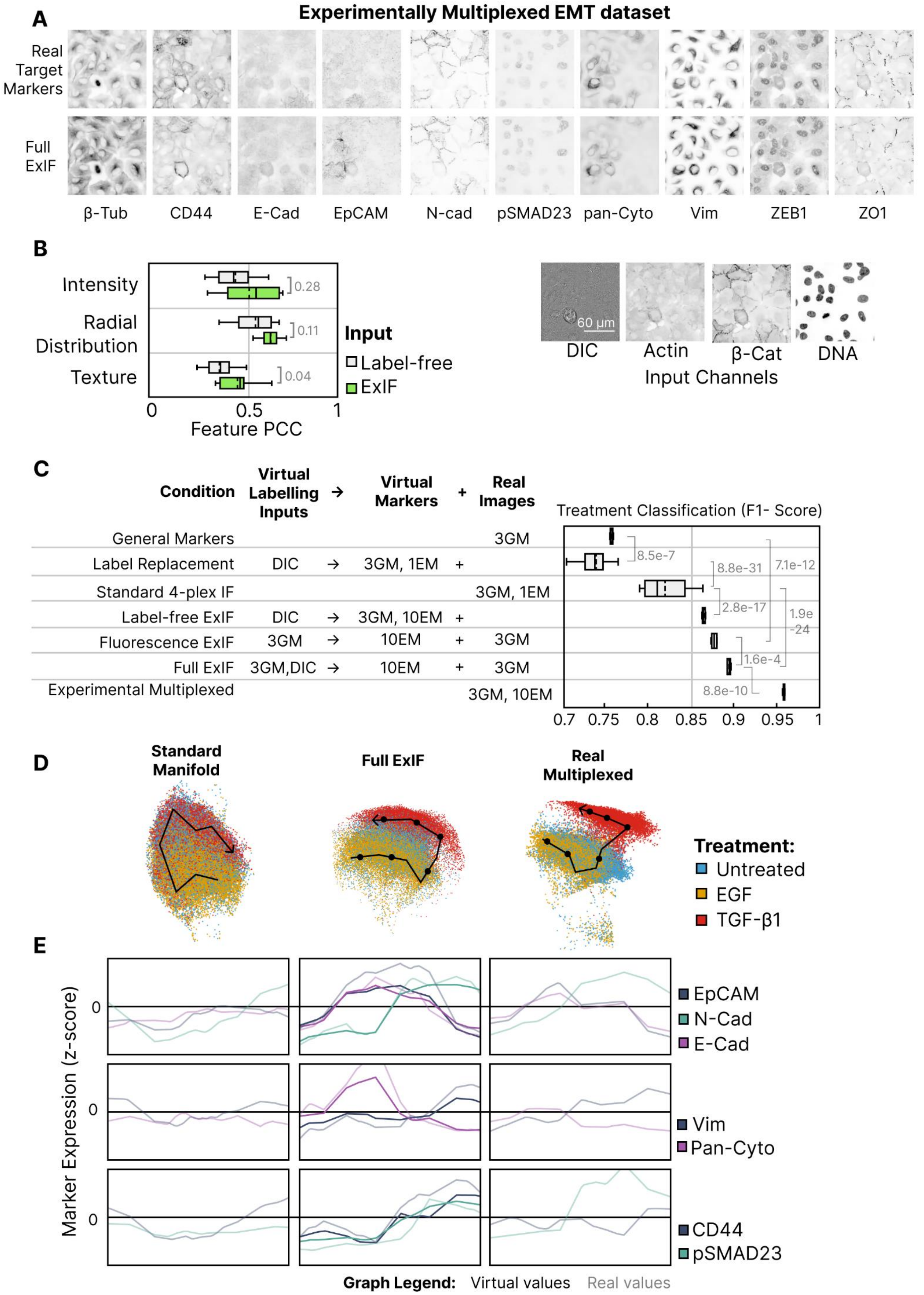

A
Experimentally Multiplexed EMT dataset
Real Target Markers
Full ExIF
β-Tub
CD44
E-Cad
EpCAM
N-cad
pSMAD23
pan-Cyto
Vim
ZEB1
ZO1
B
Intensity
Radial Distribution
Texture
0.28
0.11
0.04
0
0.5
1
Feature PCC
Input
Label-free
ExIF
60 µm
DIC
Actin
β-Cat
DNA
Input Channels
C
Condition
Virtual Labelling Inputs
Virtual Markers
Real Images
Treatment Classification (F1- Score)
General Markers
3GM
Label Replacement
DIC → 3GM, 1EM +
Standard 4-plex IF
3GM, 1EM
Label-free ExIF
DIC → 3GM, 10EM +
Fluorescence ExIF
3GM → 10EM + 3GM
Full ExIF
3GM,DIC → 10EM + 3GM
Experimental Multiplexed
3GM, 10EM
8.5e-7
8.8e-31
7.1e-12
2.8e-17
1.9e-24
1.6e-4
8.8e-10
0.7
0.75
0.8
0.85
0.9
0.95
1
D
Standard Manifold
Full ExIF
Real Multiplexed
Treatment:
Untreated
EGF
TGF-β1
E
Marker Expression (z-score)
0
EpCAM
N-Cad
E-Cad
Vim
Pan-Cyto
CD44
pSMAD23
Graph Legend: Virtual values Real values

Supplementary Figure 2. (A) Sample images shown for real experimentally labelled cells (top row) and the corresponding ExIF predictions using inputs as shown in the lower right. **(B)** 5-fold cross-validated single-cell feature-level PCC (Pearson's correlation coefficient) scores per feature category across all 10 EM markers. **(C)** 5-fold cross-validated F1-scores for SVM classification accuracy using: different real images as inputs for virtual labelling (table, left column); quantitative features from different virtual markers (table, middle column); and quantitative features from different real images (table, right column). General markers denoted by 3GM; Single variable marker denoted by 1EM; all 10 variable markers denoted by 10EM. All box plots depict data distributions as follows: dashed center line, median; solid center line, mean; box limits, upper and lower quartiles; whiskers, 1.5x interquartile range. Significance reflects Welsh's two-sided t-testing. **(D)** PHATE manifold embeddings generated using features from: the three general markers (left), the three real general markers and 10 virtual EM markers predicted by ResViT using the three general markers and DIC as input (center), or all 13 markers experimentally multiplexed (right). No cell morphological data (cell size, shape, cell-cell touching etc) was included. Black lines reflect pseudotime trajectories inferred using scFates. **(E)** Z-score normalised average EM marker intensities (y-axes) indicate EM marker dynamics relative to pseudotime (x-axes) defined in phenotypic manifolds depicted above in D showing similar trends between ExIF and experimentally multiplexed marker dynamics. In contrast, 4-plex IF reveals significantly different trends. The virtual EM marker dynamics (strong lines) also closely match real EM marker dynamics (faded lines) from the subset of cells experimentally labelled for each EM marker. Source data provided as detailed in data availability.

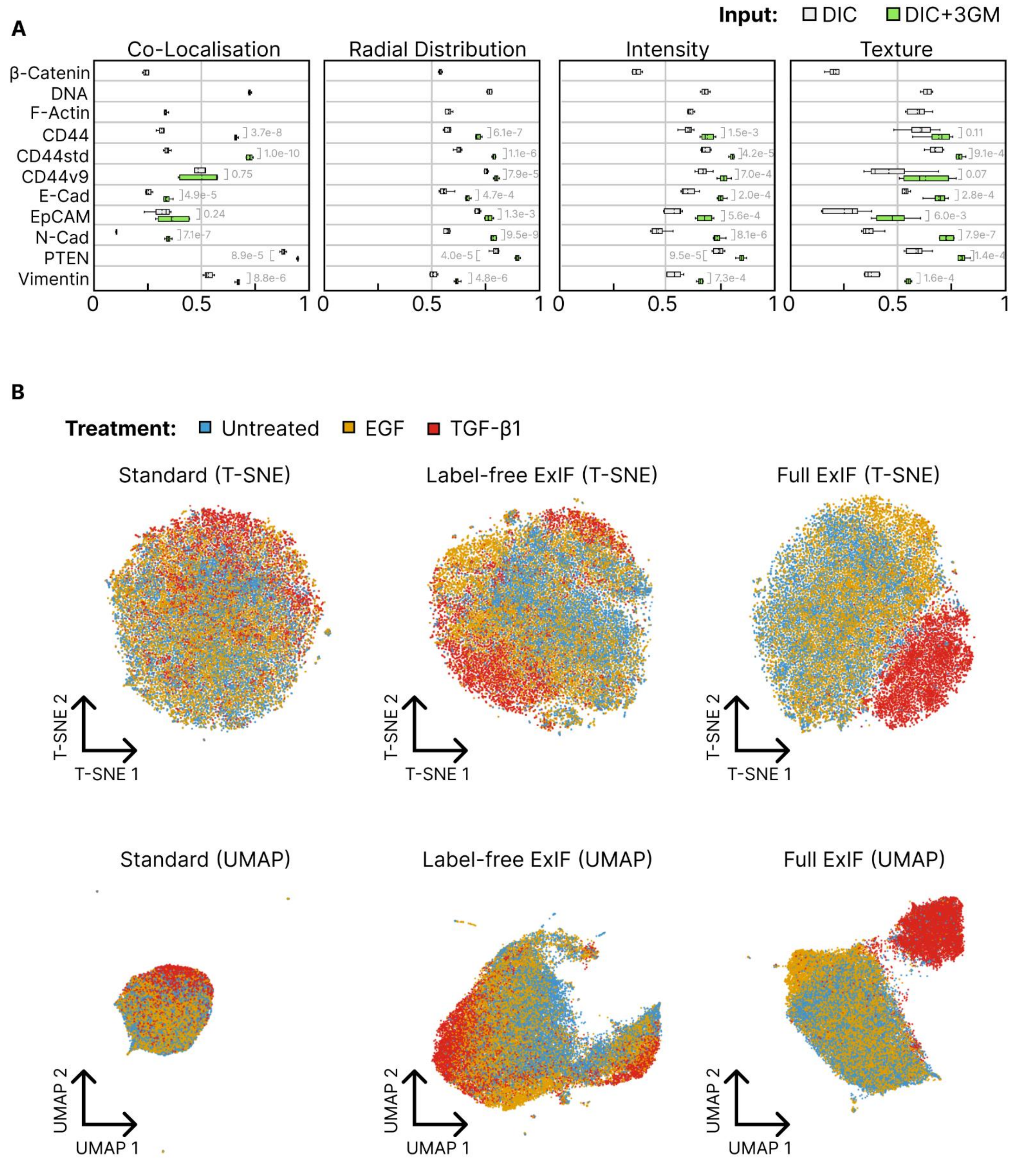

Supplementary Figure 3. (A) 5-fold cross-validated single-cell feature-level PCC (Pearson's correlation coefficient) scores per feature category, per marker; comparing virtual labelling with ResViT models receiving DIC-only input (grey) versus DIC plus the 3 general markers as concurrent model inputs (light green). Significance reflects Welsh's two-sided t-testing. (**B**) 'Standard' phenotypic manifold constructed using features extracted from the 3 real general markers only (Left). 'Label-free ExIF' phenotypic manifold constructed using features extracted from 11 virtual labels (3 common plus 8 EM markers) predicted by ResViT using only label-free (DIC) inputs (center). 'Full-ExIF' phenotypic manifold constructed using features extracted from the three real general markers and eight virtual EM markers predicted by ResViT using the three general markers and DIC as input (right). A total of 33,572 cells were analysed (12,103 control, blue; 14,350 EGF-treated, yellow; 7,119 TGF-β1-treated, red) and embedded using T-SNE (top row) or UMAP (bottom row). Source data provided as detailed in data availability.
