# Supplementary material for: Extensible Immunofluorescence (ExIF) accessibly generates high-plexity datasets by integrating standard 4-plex imaging data
Source: Nat Commun. 2025 May 17;16:4606. doi: 10.1038/s41467-025-59592-7 (PMC12085645; doi:10.1038/s41467-025-59592-7)
Supplement: Supplementary file 2 — Reporting Summary [file 41467_2025_59592_MOESM2_ESM.pdf]

nature portfolio

Corresponding author(s): Dr John Lock

Last updated by author(s): Mar 7, 2025

Reporting Summary

Nature Portfolio wishes to improve the reproducibility of the work that we publish. This form provides structure for consistency and transparency in reporting. For further information on Nature Portfolio policies, see our Editorial Policies and the Editorial Policy Checklist.

Statistics

For all statistical analyses, confirm that the following items are present in the figure legend, table legend, main text, or Methods section.

n/a	Confirmed	
☐	☒	The exact sample size (*n*) for each experimental group/condition, given as a discrete number and unit of measurement
☒	☐	A statement on whether measurements were taken from distinct samples or whether the same sample was measured repeatedly
☐	☒	The statistical test(s) used AND whether they are one- or two-sided<br>*Only common tests should be described solely by name; describe more complex techniques in the Methods section.*
☒	☐	A description of all covariates tested
☐	☒	A description of any assumptions or corrections, such as tests of normality and adjustment for multiple comparisons
☐	☒	A full description of the statistical parameters including central tendency (e.g. means) or other basic estimates (e.g. regression coefficient) AND variation (e.g. standard deviation) or associated estimates of uncertainty (e.g. confidence intervals)
☐	☒	For null hypothesis testing, the test statistic (e.g. *F*, *t*, *r*) with confidence intervals, effect sizes, degrees of freedom and *P* value noted<br>*Give P values as exact values whenever suitable.*
☒	☐	For Bayesian analysis, information on the choice of priors and Markov chain Monte Carlo settings
☒	☐	For hierarchical and complex designs, identification of the appropriate level for tests and full reporting of outcomes
☐	☒	Estimates of effect sizes (e.g. Cohen's *d*, Pearson's *r*), indicating how they were calculated

Our web collection on statistics for biologists contains articles on many of the points above.

Software and code

Policy information about availability of computer code

Data collection	Preprocessing scripts provided through github link in the main body of the manuscript.- https://doi.org/10.5281/zenodo.15172098
Data analysis	Custom code used to generate virtual labels provided through github link in the main body of the manuscript. - https://doi.org/10.5281/zenodo.15172098

For manuscripts utilizing custom algorithms or software that are central to the research but not yet described in published literature, software must be made available to editors and reviewers. We strongly encourage code deposition in a community repository (e.g. GitHub). See the Nature Portfolio guidelines for submitting code & software for further information.

Data

Policy information about availability of data

All manuscripts must include a data availability statement. This statement should provide the following information, where applicable:

- Accession codes, unique identifiers, or web links for publicly available datasets
- A description of any restrictions on data availability
- For clinical datasets or third party data, please ensure that the statement adheres to our policy

Data used in this study provided via online link via figshare - as specified in the manuscript - https://doi.org/10.6084/m9.figshare.26500210

Human research participants

Policy information about studies involving human research participants and Sex and Gender in Research.

Reporting on sex and gender	NA
Population characteristics	NA
Recruitment	NA
Ethics oversight	NA

Note that full information on the approval of the study protocol must also be provided in the manuscript.

Field-specific reporting

Please select the one below that is the best fit for your research. If you are not sure, read the appropriate sections before making your selection.

☒ Life sciences ☐ Behavioural & social sciences ☐ Ecological, evolutionary & environmental sciences

For a reference copy of the document with all sections, see nature.com/documents/nr-reporting-summary-flat.pdf

Life sciences study design

All studies must disclose on these points even when the disclosure is negative.

Sample size	Sample sizes were chosen to meet or exceed single-cell data sampling standards aimed at effectively sampling cell population heterogeneity, being in the range of 10^4 cells per condition. Moreover sample numbers reflect datasets of sufficient size to train high-fidelity deep learning models for dataset integration via virtual labelling, of which training sets all cell data were then analysed.
Data exclusions	No data was excluded
Replication	For the 8-plex experimentally multiplexed DU145 dataset (section 1), 45,780 cells were assessed from a single biological replicate with 8 technical replicates (wells). For the EMT dataset based on A549 cells (section 2), 33,572 cells were analysed from a single biological replicate across 24 distinct treatment and labelling conditions (wells). Computation experiments were based on 5-fold validation.
Randomization	Conditions applied to separate individual wells due to experimental design/constraints
Blinding	Not relevant for cell culture

Reporting for specific materials, systems and methods

We require information from authors about some types of materials, experimental systems and methods used in many studies. Here, indicate whether each material, system or method listed is relevant to your study. If you are not sure if a list item applies to your research, read the appropriate section before selecting a response.

Materials & experimental systems

n/a	Involved in the study	
☐	☒	Antibodies
☐	☒	Eukaryotic cell lines
☒	☐	Palaeontology and archaeology
☒	☐	Animals and other organisms
☒	☐	Clinical data
☒	☐	Dual use research of concern

Methods

n/a	Involved in the study	
☒	☐	ChIP-seq
☒	☐	Flow cytometry
☒	☐	MRI-based neuroimaging

Antibodies

Antibodies used	CD44,Mouse,Cell Signaling Technology,3570S<br>CD44std,Mouse,Thermo-Fischer Scientific,BMS113<br>CD44v9,Mouse,Novus Biologicals,NBP2-53204<br>COX IV,Mouse,Abcam,ab33985

	E-Cadherin,Mouse,BD Biosciences,610181<br>EpCAM,Mouse,Cell Signaling Technology,2929S<br>Fibrillarin,Rabbit,Abcam,ab5821<br>GM130,Mouse,BD Biosciences,610823<br>N-Cadherin,Mouse,Cell Signaling Technology,14215S<br>NF-kB p65,Rabbit,Cell Signaling Technology,8242S<br>PTEN,Mouse,Thermo-Fischer Scientific,32-5800<br>Vimentin,Mouse,Abcam,ab8978<br>α-Tubulin,Mouse,Abcam,ab7291<br>β-catenin,Rabbit,Thermo-Fischer Scientific,71-2700<br>4’, 6-diamidino-2-phenylindole (DAPI),N/A,Sigma-Aldrich Pty Ltd,D9532<br>Atto-647 Phalloidin,N/A,Atto-Tec GmbH,AD647-81<br>Anti-mouse IgG (H+L), F(ab’)2 Fragment (Alexa Fluor 488 Conjugate),Goat,Cell Signalling Technology,448S<br>Anti-rabbit IgG (H+L), F(ab’)2 Fragment (Alexa Fluor 555 Conjugate,Goat,Cell Signalling Technology,4413S<br><br>Conjugated Antibodies<br>E-Cadherin, Cell Signaling Technology, 3199S<br>ZEB1, Cell Signaling Technology, 29153S<br>N-Cadherin, Cell Signaling Technology, 81673S<br>Pan-Cytokeratin, Thermo-Fischer Scientific 53-9003-80<br>Actin, Abcam, ab179467<br>B-catenin, Thermo-Fischer Scientific, 71-2700/A20187<br>EpCAM, Cell Signaling Technology, 5488S<br>CD44, Cell Signaling Technology, 8724S<br>B-tubulin, Cell Signaling Technology, 3624S<br>ZO-1, Cell Signaling Technology, 98225S<br>pSMAD2/pSMAD3, BD Biosciences, 562696<br>Vimentin, BioLegend, 677807
Validation	Antibodies were selected using the AI-based BenchSci platform linking to published applications and images of each antibody in relevant use-cases (immunofluorescence labelling in the same or similar cells). Vendor images were also assessed and compared to our observed labelling patterns during in-house antibody labelling optimisation.

Eukaryotic cell lines

Policy information about cell lines and Sex and Gender in Research

Cell line source(s)	DU145 and A549 cells obtained from Ingham Institute for Applied Medical Research following mycolplasma testing (negative) and Short Tandem Repeat (STR) testing to confirm identity.
Authentication	Both DU145 and A549 cells were tested (and confirmed) for identity via Short Tandem Repeat (STR) testing based on criteria established by the ASN-0002 Standard workgroup.
Mycoplasma contamination	All cells lines are regularly tested for mycoplasma infection and tested negative throughout this project.
Commonly misidentified lines<br>(See ICLAC register)	NA
